# Leptin Levels Are Negatively Correlated with 2-Arachidonoylglycerol in the Cerebrospinal Fluid of Patients with Osteoarthritis

**DOI:** 10.1371/journal.pone.0123132

**Published:** 2015-04-02

**Authors:** James Nicholson, Syed Azim, Mario J. Rebecchi, William Galbavy, Tian Feng, Ruth Reinsel, Sabeen Rizwan, Christopher J. Fowler, Helene Benveniste, Martin Kaczocha

**Affiliations:** 1 Department of Orthopedic Surgery, Stony Brook University, Stony Brook, New York, United States of America; 2 Department of Anesthesiology, Stony Brook University, Stony Brook, New York, United States of America; 3 Department of Applied Mathematics and Statistics, Stony Brook University, Stony Brook, New York, United States of America; 4 Department of Pharmacology and Clinical Neuroscience, Umeå University, Umeå, Sweden; 5 Department of Biochemistry and Cell Biology, Stony Brook University, Stony Brook, New York, United States of America; University of Insubria, ITALY

## Abstract

**Background:**

There is compelling evidence in humans that peripheral endocannabinoid signaling is disrupted in obesity. However, little is known about the corresponding central signaling. Here, we have investigated the relationship between gender, leptin, body mass index (BMI) and levels of the endocannabinoids anandamide (AEA) and 2-arachidonoylglycerol (2-AG) in the serum and cerebrospinal fluid (CSF) of primarily overweight to obese patients with osteoarthritis.

**Methodology/Principal Findings:**

Patients (20 females, 15 males, age range 44-78 years, BMI range 24-42) undergoing total knee arthroplasty for end-stage osteoarthritis were recruited for the study. Endocannabinoids were quantified by liquid chromatography – mass spectrometry. AEA and 2-AG levels in the serum and CSF did not correlate with either age or BMI. However, 2-AG levels in the CSF, but not serum, correlated negatively with CSF leptin levels (Spearman’s ρ -0.48, P=0.0076, n=30). No such correlations were observed for AEA and leptin.

**Conclusions/Significance:**

In the patient sample investigated, there is a negative association between 2-AG and leptin levels in the CSF. This is consistent with pre-clinical studies in animals, demonstrating that leptin controls the levels of hypothalamic endocannabinoids that regulate feeding behavior.

## Introduction

The endocannabinoids anandamide (AEA) and 2-arachidonoyl glycerol (2-AG) are endogenous agonists of cannabinoid receptors and regulate a multitude of physiological processes including metabolism and food intake [1–3]. It is well-established that the endocannabinoid system plays a key role in the control of energy homeostasis and that these effects are brought about both centrally and peripherally (for reviews, see [4–7]). For example, activation of cannabinoid receptors by AEA and 2-AG promotes hyperphagia while cannabinoid receptor blockade reduces food intake and improves parameters such as fasting glucose and triglyceride levels in humans [8–13]. Leptin is a hormone produced primarily by adipose tissue and exerts its anorexigenic effects through activation of hypothalamic proopiomelanocortin neurons and downregulation of hypothalamic endocannabinoid levels [9, 14], suggesting an interplay between the central leptin and endocannabinoid systems, at least in rodents.

Previous studies have demonstrated that fasting is associated with reduced central and peripheral leptin levels in humans and elevated hypothalamic 2-AG levels in rodents [9, 15–17]. Notably, administration of leptin to rats reduces hypothalamic 2-AG levels [9], suggesting functional antagonism between the anorexigenic effects of leptin and orexigenic endocannabinoids [8]. Given this orexigenic role of the endocannabinoid system, it is perhaps not surprising that changes in the levels of the endocannabinoids and/or their synthetic or degradative enzymes have been reported in adipose tissue and plasma samples from obese, obese/diabetic patients, and patients with eating disorders [18–31]. Most studies examining central changes in endocannabinoid function have been undertaken using experimental animals following dietary intervention (see e.g. [32–34]).

Despite the documented opposing roles of endocannabinoids and leptin in the control of feeding and obesity, only one study has examined the interplay between central leptin and endocannabinoid levels in humans [28]. These authors found that 2-AG levels in the cerebrospinal fluid (CSF) were higher in an obesity-prone population (American Indians) than in Caucasians, but there was no correlation between CSF endocannabinoid and leptin levels [28]. In a recent study, we investigated associations between pain and endocannabinoid levels in the serum and CSF from elderly, overnight fasted overweight/obese individuals suffering from osteoarthritis [35]. In the present study, we have investigated the relationship between central and peripheral endocannabinoid levels, body-mass index (BMI), and leptin levels in these individuals.

## Materials and Methods

### Ethics Statement

The experiments conducted herein were approved by the Stony Brook University institutional review board and written consent was obtained from each patient.

### Study participants

Total knee arthroplasty (TKA) patients (n = 35) scheduled for an elective unilateral TKA under spinal anesthesia and a femoral nerve block were enrolled for this study. Patients with documented rheumatoid arthritis, patients scheduled for bilateral TKA, and patients scheduled for a TKA revision were excluded from the current study. Details of the patient population used here are given in [35].

### Experimental design & data collection

Immediately prior to the surgery, at the time of the spinal anesthesia, blood and CSF were collected from fasting (10–18 hours) patients.

### Analysis of leptin levels

Blood was drawn from patients into tubes lacking anticoagulant (BD Vacutainer) and the resulting serum samples prepared, flash frozen, and stored at -80°C until use. CSF was collected and subjected to centrifugation at 1800 x g for 15 min at 4°C and the resulting supernatant was flash frozen in liquid nitrogen and stored at -80°C. Leptin levels were quantified using a leptin enzyme-linked immunosorbent assay (Quantikine immunoassay, R&D Systems). The samples were diluted by gender as suggested by the manufacturer. Each sample was assayed in triplicate using the protocol supplied by the manufacturer. The color intensities were read at 450 nm.

### Quantification of Endocannabinoids

Serum and CSF (0.5 ml) were mixed with 3.5 ml of 2:1:1 CHCl3:MeOH:Tris (50 mM, pH 8) that was spiked with 4 ng d_4_-PEA, d_2_-OEA, d_5_-2-AG, and 400 pg d_4_-AEA. Following centrifugation, the organic layer was removed and the sample was extracted again with the same buffer. The organic layer was dried down under argon and resuspended with 120 μl of 2:1 CHCl_3_:MeOH and 10 μl was injected into a Thermo TSQ Quantum Access Triple Quadropole mass spectrometer in triplicate. Endocannabinoid quantification was performed exactly as described [35, 36].

### Statistical analysis

Zero-order correlation coefficients were determined using the statistical package built into the GraphPad Prism computer program for the Macintosh (GraphPad Software Inc., San Diego, CA, USA). First-order Spearman’s ρ values were calculated from the corresponding zero-order (bivariate) values using the formula of Lehmann [37]. Multiple linear regression analyses were conducted using using the IBS SPSS Statistics package, version 22. The rank-based two-way ANOVAs were calculated using the function raov in the Rfit package of the R computer program [38, 39]. We consider a p-value less than 0.05 as statistically significant.

## Results

### Endocannabinoid levels in the patient samples

The characteristics of the collective sample, stratified by gender and diagnosis of diabetes, are shown in Table 1. Given that the sample sizes are small, particularly for the diabetic subjects, the data are presented as medians and ranges, and a non-parametric rank-based two-way ANOVA [38] was used to assess significance. The body mass index (BMI) was higher in the diabetics vs. non-diabetics. As expected [40, 41], both serum and CSF leptin levels were significantly higher in the females than the males. The two-way ANOVAs confirmed these main effects and also found a significant interaction between gender and diabetes for the serum, but not CSF leptin (Table 1). Among the endocannabinoids, there were no significant main effects of either gender or diabetes, or interactions between gender and diabetes (Table 1).

**Table 1:** Characteristics of the study group stratified on the basis of gender (G) and incidence of diabetes (D).

	**Diabetes**	**Females**	**Males**	**P value**
Age (years)	no	68 (44–76, 14)	70 (55–78, 11)	G: 0.44
	yes	64 (51–78, 6)	67 (59–70, 4)	D:0.30
				GxD:0.91
BMI (kg/m^2^)	no	34 (26–42, 14)	28 (24–35, 11)	G: 0.066
	yes	36 (29–40, 6)	36 (33–39, 4)	**D:0.002**
				GxD:0.064
Serum leptin	no	45 (16–85, 14)	9.3 (4.3–18, 9)	**G: 0.0002**
	yes	36 (14–56, 6)	28 (16–32, 3)	D:0.43
				**GxD:0.0095**
CSF leptin	no	290 (180–440, 14)	140 (42–190, 9)	**G: 0.0005**
	yes	240 (190–390, 6)	210 (110–220, 3)	D:0.87
				GxD:0.18
Serum AEA	no	0.26 (0.11–0.54, 14)	0.21 (0.11–0.33, 11)	G: 0.082
	yes	0.28 (0.13–0.31, 5)	0.15 (0.092–0.15, 4)	D:0.41
				GxD:0.59
Serum 2-AG	no	3.4 (0.91–6.1, 14)	3.5 (0.96–5.0, 11)	G: 0.23
	yes	3.3 (0.67–5.5, 6)	1.4 (0.86–2.9, 4)	D:0.23
				GxD:0.31
CSF AEA	no	14 (6–22, 14)	17 (4–30, 11)	G: 0.96
	yes	11 (8–25, 5)	10 (6–21, 4)	D:0.38
				GxD:0.33
CSF 2-AG	no	64 (30–220, 13)	99 (14–170, 11)	G: 0.45
	yes	99 (0–180, 5)	100 (75–120, 4)	D:0.84
				GxD:0.76

Data are given as medians with ranges, followed by the sample sizes in parentheses. Serum leptin levels are given as ng/ml while CSF leptin levels are given as pg/ml; serum AEA and 2-AG levels are given as ng/ml; and CSF AEA and 2-AG levels are given as pg/ml. P values are calculated using a non-parametric two-way robust Wilcoxon analysis [38]. Significant correlations are shown in boldface.

### Correlation between leptin and endocannabinoid levels

**Fig 1:**
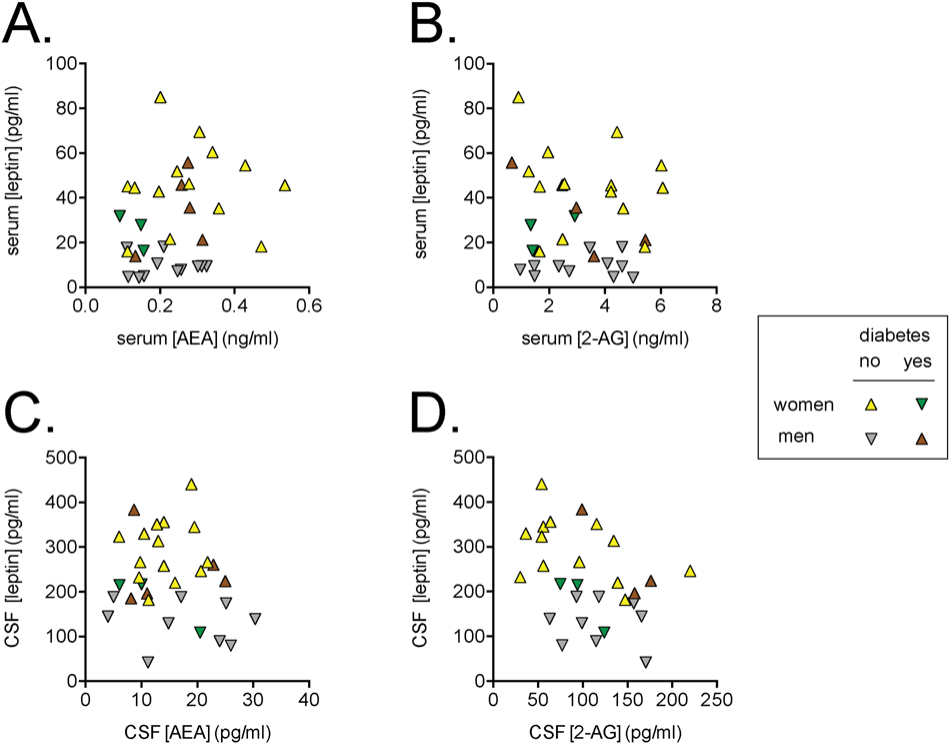
Relationship between endocannabinoid and leptin levels in the serum (A, B) and CSF (C, D). Shown are the data for AEA (Panels A, C) and 2-AG (Panels B, D). The Spearman ρ values are provided in Table 2.

Bivariate correlations were determined between the outcome parameters used in the present study. Before undertaking such correlations, it is important to determine whether the data are normally distributed because a parametric Pearson correlation is not appropriate if this is not the case. We used the D'Agostino & Pearson omnibus normality test and found that the serum 2-AG values were not normally distributed (all other variables reported passed this test). Consequently, we used non-parametric Spearman’s correlations for the whole dataset. The correlation coefficients for the endocannabinoids and for leptin are shown in Table 2, and the scatterplots for the endocannabinoids against the corresponding leptin concentrations are shown in Fig 1. As expected from the literature [15], CSF and serum leptin concentrations were highly correlated (Spearman’s ρ = 0.83, n = 31, P<0.0001) and thus it is not surprising that the significant association of BMI with leptin was seen for both serum and CSF leptin. In their original study, Schwartz et al. [41] reported that the best correlation for CSF leptin as dependent variable was seen in a model incorporating gender, plasma leptin and log plasma leptin as independents. However, this is likely to result in multicollinearity (a potential source of misleading results when one or more of the predictor variables are highly correlated, i.e. not independent), and this was indeed seen in our dataset. A simpler model with gender and either unlogged serum or logged serum leptin (but not both) gave similar r^2^ values (0.69 and 0.71) with both parameters contributing significantly, and the lowest level of collinearity (variance inflation factor value 1.8; as a rule of thumb, a value ≥5 indicates multicollinearity) was seen with the unlogged values (data not shown).

**Table 2:** Correlations between endocannabinoid and leptin concentrations and the corresponding parameters for age, BMI, AEA and 2-AG.

**Parameter 1**	**Parameter 2**	**Serum**	**CSF**
Age	Leptin	-0.20 (P = 0.26, n = 33)	-0.015 (P = 0.94, n = 32)
	AEA	-0.012 (P = 0.95, n = 34)	-0.058 (P = 0.75, n = 34)
	2-AG	0.042 (P = 0.81, n = 35)	-0.27 (P = 0.13, n = 33)
BMI	Leptin	**0.65 (P<0.0001, n = 33)**	**0.49 (P = 0.0048, n = 32)**
	AEA	0.18 (P = 0.30, n = 34)	-0.031 (P = 0.86, n = 34)
	2-AG	-0.13 (P = 0.47, n = 35)	-0.019 (P = 0.91, n = 33)
Leptin	AEA	0.29 (P = 0.10, n = 33)	-0.17 (P = 0.35, n = 31)
	2-AG	-0.09 (P = 0.62, n = 33)	**-0.48 (P = 0.0076, n = 30)**
AEA	2-AG	0.18 (P = 0.31, n = 34)	0.13 (P = 0.47, n = 32)

Values are Spearman’s ρ, in deference to the non-normal distribution of serum 2-AG in the sample. The other parameters passed the D'Agostino & Pearson omnibus normality test. Significant correlations are shown in boldface.

There was no significant association between AEA and the corresponding leptin levels. As reported previously for this patient population [35], serum AEA and CSF AEA levels were significantly (p < 0.05) correlated. No such correlation between serum and CSF 2-AG levels was seen or between serum 2-AG and serum leptin levels (Table 2). However, a significant negative correlation (ρ = -0.48, n = 30, P = 0.0076) was observed between CSF 2-AG and leptin levels (Table 2, Fig 1).

To exclude the possibility that the negative correlation between leptin and 2-AG was a result of an underlying distribution of another significantly associated parameter, higher-order correlations controlling for additional parameters were applied. The first order Spearman’s ρ value for CSF leptin vs. CSF 2-AG controlling for BMI, remained significant (ρ = -0.50, n = 30, P = 0.0059). Given that both the CSF leptin and the CSF 2-AG values passed the D'Agostino & Pearson omnibus normality test, it is possible to explore the relationship between these two variables further in multiple regression analyses. The statistical program we used allows different approaches for how the independent variables are presented. We used backward elimination (where all the candidate variables are initially included, and variables are removed when such deletion improves the model), with CSF 2-AG as the dependent variable and with age, gender, incidence of diabetes, BMI and CSF leptin as the independent variables. Using this approach, the only significant variable remaining in the final model was CSF leptin, and the same was true when forward selection (when variables are added to the model) was tested. Given that the data indicate an association rather than causality, the reverse analysis, i.e. where CSF leptin was the dependent variable, was also investigated. Both methods selected CSF 2-AG and diabetes as significant predictors. The backward elimination analyses are summarized in S1 and S2 Tables. Thus, the association between CSF leptin and 2-AG levels found here is not induced by the other parameters investigated.

## Discussion

The endocannabinoid system regulates appetite and metabolism [8]. Previous work in rodents suggests an antagonistic relationship between the central endocannabinoid system and leptin [9]. Indeed, administration of leptin to normal Sprague-Dawley rats reduced hypothalamic levels of both AEA and 2-AG [9]. Obese Zucker (fa/fa) rats with defective leptin signaling possess higher hypothalamic 2-AG levels than lean controls, whereas AEA levels are not changed. Elevated hypothalamic 2-AG levels are observed in ob/ob mice (where there is no leptin production) compared to controls. Furthermore, fasting elevates while feeding reduces hypothalamic 2-AG levels in rats [16] and fasting results in reduced leptin levels in the CSF of humans [15, 17]. Based upon these studies, fasting in humans would be expected to produce an inverse gradient between CSF 2-AG and leptin levels. Indeed, such a negative correlation between 2-AG and leptin was observed in the current study, suggesting that the control of 2-AG production by leptin seen in the rodent hypothalamus [9] may also occur in humans.

Leptin is an adipokine that regulates appetite, metabolism, and inflammation and one of its primary roles is to reduce food intake [42–44]. Obesity is associated with chronically elevated leptin levels that may give rise to leptin resistance [45]. Recent work has shown that peripheral endocannabinoid signaling contributes to leptin resistance [11]. In contrast, lack of hypothalamic endocannabinoid signaling leads to leptin resistance in animals fed a high fat diet, whereas its actions are potentiated in chow-fed animals [46]. In our study, the negative correlation between CSF leptin and 2-AG suggests that the reciprocal regulation of central leptin and endocannabinoid signaling remains functionally coupled in overweight fasting humans. Because our serum and CSF samples were obtained during the morning hours, it is tempting to speculate that the inverse relationship between leptin and 2-AG may reflect an increase in the morning orexigenic drive. This is supported by a very recent clinical study demonstrating that 2-AG levels increase while leptin levels decrease during sleep and throughout the morning [47].

Our results are in contrast to the study of Jumpertz et al [28], who did not observe a correlation between leptin and 2-AG levels in the CSF of healthy, middle aged overweight individuals, which the authors attributed to possible leptin resistance in this cohort. Although our sample comprised older patients with osteoarthritis, it is unlikely that the endocannabinoid and leptin levels were affected by the underlying osteoarthritis (see below). However, it is prudent to consider the present findings in context of this co-morbidity and again to suggest that further validation studies are needed. Lastly, the negative correlation between CSF 2-AG and leptin in the fasting subjects would infer that sufficient 2-AG levels derived from the hypothalamus are reflected in the CSF for the effect to be specific to the hypothalamic endocannabinoid signaling process.

Importantly, the CSF endocannabinoid levels reported herein are in line with the literature (Table 3). Most of the previously reported data are for AEA, with mean values for controls ranging from below the limit of detection to 11.65±7.53 pmol/ml (Table 3). Similarly, 2-AG levels ranging from below the limit of detection to 160±110 pmol/mg (61±42 ng/ml) have been reported; and our values are within this range. Lastly, the CSF leptin levels reported herein are similar to those previously observed in subjects without underlying osteoarthritis [48, 49], arguing against osteoarthritis-induced alterations in leptin levels.

**Table 3:** Literature values of AEA and 2-AG (+1-AG) levels in the CSF.

**Study**	**Control/Patients**	**n**	**AEA**	**2-AG**
Giuffrida et al. [50]	Controls	81	0.007±0.002^b^	
	Schizophrenic patients: Antipsychotic-naïve	24	0.057±0.011^b^	
	1 °G antipsychotic treated	36	0.031±0.012^b^	
	2 °G antipsychotic treated	31	0.062±0.016^b^	
Pisani et al [51]	Controls	14	5.23±1.81^a^	
	Untreated PD patients	16	10.7±3.2^a^	
Sarchielli et al. [52]	Controls	20	0.39±0.09^b^	Below detection limit (0.2 pmol/sample) for all groups
	Chronic migraine	15	0.21±0.06^b^	
	Probable migraine + PAOH	15	0.22±0.05^b^	
Centonze et al. [53]	Controls	11	2.4±1.3	~75–100^c^
	Relapsing-remitting MS	11	20.3±15.7	
Leweke et al. [54]	Controls	81	range 0 - ~0.07^c^	
	Schizophrenics	44	range 0 - ~0.33^c^	
Di Filippo et al. [55]	Controls	20	0.085±0.0019^a^	1.068±0.123^a^
	Stable remitting-relapsing MS	20	0.0048±0.0009^a^	0.824±0.104^a^
	MS during relapse	15	0.0068±0.0009^a^	0.997±0.127^a^
	Secondary phase MS	15	0.0036±0.0006^a^	0.762±0.098^a^
Koethe et al. [56]	Controls	81	Median <0.001, IQR <0.001–0.005	
	Prodromal psychotic patients	27	Median 0.006, IQR <0.001–0.073	
Koppel et al. [57]	Elderly controls and Late onset AD patients	35	Below detection limit	For all cases mean 0.205, range 0.027–0.704
Koethe et al. [58]	Controls before and after sleep deprivation	20	Very low levels shown in Fig 1 of paper	
Romigi et al. [59]	Controls	9,6^d^	11.65±7.53^a^	160±110^a^
	Untreated epilepsy	9,6^d^	2.55±1.78^a^	210±147^a^
Pisani et al. [60]	Controls	37	4.59±1.65^a^	
	De novo PD	38	9.76±3.26^a^	
	PD, treatment withdrawal	8	11.19±3.23^a^	
	PD, under treatment	10	6.76±3.41^a^	
Jumpertz et al. [28]	Controls (BMI 34±8^a^ kg/m^2^)	27	0.06±0.08^a^	14.2±6.85^a^
Morgan et al. [61]	Controls	13	~0.13^c^	~10^c^
	Light cannabis users	10	~0.16^c^	~18^c^
	Heavy cannabis users	10	~0.10^c^	~23^c^

^a^Means ± SD;

^b^means ±SE;

^c^values not given explicitly in the text but estimated from the figures;

^d^refers to samples analysed for AEA and 2-AG, respectively.

Abbreviations: 1 °G and 2 °G, first- and second-generation, respectively; AD, Alzheimer’s disease; IQR, interquartile range; MS, multiple sclerosis; PAOH, probable analgesic overuse headache; PD, Parkinson’s disease.

All values are in pmol/ml.

One observation at odds with the literature, however, is the finding of a significant correlation between serum and CSF AEA (but not 2-AG) levels. In four separate studies [28, 50, 56, 61] no such correlation was found, although a positive correlation was seen for schizophrenic patients treated with first-generation antipsychotics [50]. Very recently completed data (i.e. after this manuscript was submitted) by one of us suggests that there is a large inter-sample variation in plasma AEA and 2-AG levels for any given individual when the samples are either taken at a one hour interval or at the same time of day on two different occasions (L. Lindgren, M. Gouveia-Figueira, M.L. Nodding and Christopher J. Fowler, manuscript in preparation). This may account for the differences in plasma:CSF correlation coefficients between the studies. Additionally, it may be possible that in the patient population examined here, the underlying osteoarthritis has produced an effect upon AEA turnover centrally and/or peripherally, thereby inducing a correlation. There is evidence that both inflammatory and anti-inflammatory cytokines can affect components of the endocannabinoid system, such as cannabinoid receptors and fatty acid amide hydrolase [62–64] so in theory this is possible although unlikely because the serum endocannabinoid levels obtained here are similar to those reported for healthy patients without underlying osteoarthritis [65–67]. Furthermore, brain and plasma AEA levels were found not to be altered in an animal model of osteoarthritis [68].

A second potential factor to consider may be the body weight range of the patient sample. In our dataset, only 2 individuals were classified as normal weight (BMI<25) whilst 9 were overweight (25<BMI<30), 22 were obese (30<BMI<40) and 2 were morbidly obese BMI>40). Thus, our findings relate to an overweight/obese population. This may be of importance for the observed and interpreted results, although the literature is somewhat divergent with respect to the association between circulating endocannabinoid levels and body weight / BMI. Indeed, some studies have reported elevated levels of both AEA and 2-AG in overweight and obese individuals [18, 22], some have reported elevations in only one of the endocannabinoids [19, 21, 28, 31], while others have reported no change in either metabolite [24]. The only study investigating the CSF was that of Jumpertz et al. [28], who reported a negative correlation between BMI and AEA, but not 2-AG, levels; but this association was lost in a multivariate analysis adjusting for plasma AEA concentrations. However, our data reveal that central and circulating endocannabinoid levels do not correlate with BMI at least in an osteoarthritis patient population.

In conclusion, the present study investigated the relationship between obesity factors and peripheral and central endocannabinoids in a population of overweight to obese individuals with severe osteoarthritis. Because endocannabinoid levels are dynamically regulated by feeding and fasting in rodents and humans [16, 20, 69], it is clear that the relationship between circulating and central endocannabinoids and obesity-related parameters is complex and most likely dependent upon the patient populations selected. Therefore, it should be noted that the conclusions of our study are restricted to this patient category and some of the differences between the present study and other published data for other patient categories may reflect this fact. Nonetheless, the present study provides evidence for the tight coupling between central endocannabinoid and leptin levels and suggests that the regulation of 2-AG levels by leptin observed in the rodent hypothalamus may likewise exist in humans.

## Supporting Information

S1 Table Multiple linear regression analysis with backward elimination for CSF 2-AG as dependent variable, and age (A), BMI (W), gender (G), incidence of diabetes (D) and CSF leptin (L) as independent variables. B in the table refers to the unstandardized coefficients, and these are only shown for analyses with significant ANOVA values.
(DOCX)

S2 Table Multiple linear regression analysis with backward elimination for CSF leptin as dependent variable, and age (A), BMI (W), gender (G), incidence of diabetes (D) and CSF 2-AG (2AG) as independent variables. B in the table refers to the unstandardized coefficients, and these are only shown for CSF 2-AG, gender and the constant; the other variables did not reach significance in any case (P > 0.3).
(DOCX)
