# Supplementary material for: Leptin Levels Are Negatively Correlated with 2-Arachidonoylglycerol in the Cerebrospinal Fluid of Patients with Osteoarthritis
Source: PLoS One. 2015 Apr 2;10(4):e0123132. doi: 10.1371/journal.pone.0123132 (PMC4383333; doi:10.1371/journal.pone.0123132)
Supplement: S1 Table — B in the table refers to the unstandardized coefficients, and these are only shown for analyses with significant ANOVA values. (DOCX) [file pone.0123132.s001.docx]

**S1 Table.** Multiple linear regression analysis with backward elimination for CSF 2-AG as dependent variable, and age (A), BMI (W), gender (G), incidence of diabetes (D) and CSF leptin (L) as independent variables. B in the table refers to the unstandardized coefficients, and these are only shown for analyses with significant ANOVA values.

| **Model** | **r^2^** | **ANOVA** | **Unstandardized B (±SE)** | **P value** |
| --- | --- | --- | --- | --- |
| 1 (A,W,G,D,L) | 0.27 | F_5,24_=1.81, P=0.149 |  |  |
| 2 (A,G,D,L) | 0.27 | F_4,25_=2.30, P=0.086 |  |  |
| 3 (A,G,L) | 0.26 | F_3,26_=3.12, P=0.043 | (Constant) 262±77 | 0.0020 |
|  |  |  | Age -1.10±1.07 | 0.31 |
|  |  |  | Gender -26.0±28.4 | 0.37 |
|  |  |  | CSF leptin -0.34±0.14 | 0.020 |
|  |  |  |  |  |
| 4 (A,L) | 0.24 | F_2,27_=4.28, P=0.024 | (Constant) 244±74 | 0.0026 |
|  |  |  | Age -1.33±1.04 | 0.21 |
|  |  |  | CSF leptin -0.25±0.09 | 0.011 |
|  |  |  |  |  |
| 5 (L) | 0.19 | F_1,28_=6.76, P=0.015 | (Constant) 155±23 | <0.0001 |
|  |  |  | CSF leptin -0.24±0.09 | 0.015 |

Gender was coded as dummy variables female = 0, male = 1. Diabetes was coded as dummy variables no = 0, yes = 1. The adjusted r^2^ values for models 1-5 were 0.12, 0.15, 0.18, 0.18 and 0.17, respectively. In all cases, the VIF values were <2.6. Models 3 and 5 were further investigated, and visual inspection of the standardized predicted value *vs.* standardized residual suggested no overt heteroscedasticity or the presence of outliers. The Breusch-Pagan test (where the null hypothesis is homoscedasticity) gave P values of 0.57 and 0.88 for models 3 and 5, respectively.
