# Supplementary material for: Leptin Levels Are Negatively Correlated with 2-Arachidonoylglycerol in the Cerebrospinal Fluid of Patients with Osteoarthritis
Source: PLoS One. 2015 Apr 2;10(4):e0123132. doi: 10.1371/journal.pone.0123132 (PMC4383333; doi:10.1371/journal.pone.0123132)
Supplement: S2 Table — B in the table refers to the unstandardized coefficients, and these are only shown for CSF 2-AG, gender and the constant; the other variables did not reach significance in any case (P > 0.3). (DOCX) [file pone.0123132.s002.docx]

**S2 Table.** Multiple linear regression analysis with backward elimination for CSF leptin as dependent variable, and age (A), BMI (W), gender (G), incidence of diabetes (D) and CSF 2-AG (2AG) as independent variables. B in the table refers to the unstandardized coefficients, and these are only shown for CSF 2-AG, gender and the constant; the other variables did not reach significance in any case (P > 0.3).

| **Model** | **r^2^** | **ANOVA** | **Unstandardized B (±SE)** | **P value** |
| --- | --- | --- | --- | --- |
| 1 (A,W,G,D,2AG) | 0.67 | F_5,24_=9.68, P<0.0001 | (constant) 225±162 | 0.18 |
|  |  |  | gender -130±28 | <0.0001 |
|  |  |  | CSF 2-AG -0.55±0.23 | 0.026 |
|  |  |  |  |  |
| 2 (A,W,G,2AG) | 0.67 | F_4,25_=12.6, P<0.0001 | (constant) 229±154 | 0.15 |
|  |  |  | gender -131±27 | <0.0001 |
|  |  |  | CSF 2-AG -0.55±0.23 | 0.023 |
|  |  |  |  |  |
| 3 (W,G,2AG) | 0.67 | F_3,26_=17.4, P<0.0001 | (constant) 266±93 | 0.0082 |
|  |  |  | gender -130±26 | <0.0001 |
|  |  |  | CSF 2-AG -0.57±0.22 | 0.015 |
|  |  |  |  |  |
| 4 (G,2AG) | 0.66 | F_2,27_=25.8, P<0.0001 | (constant) 347±24 | <0.0001 |
|  |  |  | gender -140±23 | <0.0001 |
|  |  |  | CSF 2-AG -0.57±0.22 | 0.014 |

For coding of dummy variables, see Legend to Table S1. The adjusted r^2^ values for models 1-4 were 0.60, 0.62, 0.63 and 0.63, respectively. In all cases, the VIF values were <1.8. Model 4 was further investigated, and visual inspection of the standardized predicted value *vs.* standardized residual suggested no overt heteroscedasticity or the presence of outliers. The Breusch-Pagan test (where the null hypothesis is homoscedasticity) gave a P value of 0.32.
